# Supplementary material for: Antibiotic treatment duration for bloodstream infections in critically ill children—A survey of pediatric infectious diseases and critical care clinicians for clinical equipoise
Source: PLoS One. 2022 Jul 26;17(7):e0272021. doi: 10.1371/journal.pone.0272021 (PMC9321425; doi:10.1371/journal.pone.0272021)
Supplement: S1 File — (PDF) [file pone.0272021.s001.pdf]

**Antibiotic treatment duration for bloodstream infections in critically ill children--a survey of pediatric infectious diseases and critical care clinicians**

**Principal investigators**: Nick Daneman MD MSc, Sandra Pong PharmD

**Co-investigators**: Robert Fowler MDCM MS (Epi), Asgar Rishu MBBS, Michelle Science MD MSc, James S Hutchison MD, Elaine Gilfoyle MD MMEd, Winnie Seto PharmD MSc, Srinivas Murthy MDCM MHSc, Philippe Jouvet MD PhD, Jeffrey Pernica MD MSc, Patricia Fontela MD PhD, Nicholas Mitsakakis MSc PhD PStat

**Institution**: Sunnybrook Health Sciences Centre

---

Dear Survey Respondent,

You are invited to participate in a confidential, online, web-based survey for research to study antibiotic treatment durations for confirmed bloodstream infections in critically ill children.

If you decide to participate, you will be presented with 5 brief patient scenarios involving children with bloodstream infections admitted to the pediatric intensive care unit (PICU). The goal is to learn about the usual duration of antibiotic therapy that are prescribed for these patients.

After the patient scenarios, you will be asked 1 question about extrapolating data from an adult trial to pediatric patients, 1 question about interpreting data from a pediatric trial[1] and 1 more question about what trial outcomes you think are important in PICU patients.

This survey takes approximately 10 minutes to complete.[2]

Link to survey: (insert survey link)

If you choose to complete the survey, this will constitute your consent to participate in the study. Any information that you provide is confidential and any personal information will be de-identified. All data will be summarized, and no individual person will be identified from the results. The data collected from this study will be maintained on a password-encrypted computer database without personal identifiers, archived electronically after study completion, maintained for 10 years and then destroyed afterwards.

Participation in this study is voluntary and you may decline to answer any questions by leaving them blank. You may withdraw your participation at any time without consequences by closing the survey.[3] There are no known or anticipated risks from participating in this study.

Please note that the online survey is hosted by "Survey Monkey" which is a web survey company located in the USA. All responses to the survey will be stored and accessed in the USA. This company is subject to U.S. laws, in particular, to the U.S. FREEDOM Act that allows authorities access to the records of internet service providers. If you choose to participate in the survey you understand that your responses to the questions and your IP address will be stored and accessed in the USA. The security and privacy policy for Survey Monkey can be viewed at http://www.surveymonkey.com/

If you have any questions about this study, you may contact Sandy Pong (sandra.pong@sickkids.ca) or Dr. Nick Daneman (nick.daneman@sunnybrook.ca)

The Sunnybrook Research Ethics Board has reviewed this study. If you have questions about your rights as a research participant or any ethical issues related to this study that you wish to discuss with someone not directly involved with the study, you may call the Chair of the Sunnybrook Research Ethics Board at (416) 480-6100 ext. 88144.

Thank you very much for your consideration to participate in this survey study.

Sincerely,

Dr. Nick Daneman, MD, MSc, FRCPC
Clinician Scientist
Division Head Infectious Diseases
Sunnybrook Health Sciences Centre, University of Toronto

Sandra Pong, PharmD
Graduate student, Institute of Health Policy, Management and Evaluation, University of Toronto
Clinical Pharmacist, The Hospital for Sick Children

**Introduction**
In this survey, you will be presented with 5 brief patient scenarios involving children with bloodstream infections admitted to the pediatric intensive care unit (PICU). The goal is to learn about the usual duration of antibiotic therapy that are prescribed for these patients.

After the patient scenarios, you will be asked 1 question about extrapolating data from an adult trial to pediatric patients, 1 question about interpreting data from a pediatric trial[1] and 1 more question about what trial outcomes you think are important in PICU patients.

**General demographics**
* 1. What is your medical specialty? (Check all that apply)
   - Critical care physician
   - Infectious diseases physician
   - Pharmacist
   - Nurse practitioner
   - Fellow/trainee enrolled in a clinical training program
   - Other (please specify)

* 2. In your primary clinical practice, do you primarily treat:
   - Pediatric patients only (age <18 years)
   - Adult patients only (age >18 years)
   - Both pediatric and adult patients

* 3. Where is your practice location? (Check all that apply)
   - Canada
   - Australia
   - New Zealand
   - Other (please specify)

* 4. Select the most appropriate description of your primary clinical practice location
   - Teaching hospital
   - Non-teaching hospital
   - Other (please specify)

* 5. How many years have you been in practice since graduating from medical school or equivalent?
   - 0-5
   - 6-10
   - 11-15
   - 16-20
   - ≥21

* 6. What is the approximate number of patients with bloodstream infections that you treat each year?
   - 0-10
   - 11-20
   - ≥21

* 7. Does your practice site have an active antimicrobial stewardship program?
  - o Yes
  - o No
  - o Not sure

* 8. Are you a member of the antimicrobial stewardship program?
  - o Yes
  - o No

**Patient 1**
A 7-year old male presents to the Emergency Department with fever, respiratory distress and hypotension. He has a history of cerebral palsy and developmental delay.

He receives early resuscitation including vasopressor support. Blood and urine cultures are sent, he is started empirically on ceftriaxone and vancomycin and transferred to the PICU. On admission to the ICU, he is intubated and bronchoalveolar lavage (BAL) cultures are sent. Over the next 24 hours, his vasopressor requirements are weaned and then discontinued.

At 48 hours, his BAL and blood cultures have grown *Streptococcus pneumoniae*. His urine culture is negative. He is diagnosed with bloodstream infection secondary to pneumonia.

* 9. Based on the information above, what total duration (in days) of antibiotic therapy would you usually/typically recommend for this patient?
  - o Number of days

* 10. Would you be willing to enrol a patient like this into a study comparing 7 days vs. 14 days of antimicrobial therapy to treat bacteremia?
  - o Yes
  - o No

**Patient 2**
A 9-month old male has been admitted to the PICU for the past 2 weeks. He currently remains on non-invasive positive pressure ventilation. He also has significant GERD, failure to thrive and is total parenteral nutrition (TPN)-dependent.

Today, he develops a new onset fever of 38.8 C and increased work of breathing. His chest radiograph shows no collapse or consolidation. Blood (central and peripheral) and urine cultures are drawn, and the patient is started on ceftriaxone and vancomycin.

At 12 hours, the patient's blood culture becomes positive for *Enterococcus faecalis*. His urine culture is negative. An echocardiogram is ordered and reported negative. He is diagnosed with central-venous catheter associated bloodstream infection. The central line is removed.

* 11. Based on the information above, what total duration (in days) of antibiotic therapy would you usually/typically recommend for this patient?
  - o Number of days

* 12. What total duration (in days) of antibiotic therapy would you usually/typically recommend if the central line was not removed?
  - Number of days

* 13. If the line was removed, what total duration (in days) would you usually/typically recommend if the blood culture grew:
  - *Staphylococcus aureus*
  - *Klebsiella pneumoniae*
  - Coagulase negative staphylococci
  - *Escherichia coli*
  - *Enterobacter cloacae*
  - *Pseudomonas aeruginosa*

* 14. If the line was not removed, what total duration (in days) would you usually/typically recommend if the blood culture grew:
  - *Staphylococcus aureus*
  - *Klebsiella pneumoniae*
  - Coagulase negative staphylococci
  - *Escherichia coli*
  - *Enterobacter cloacae*
  - *Pseudomonas aeruginosa*

* 15. Would you be willing to enrol a patient like this into a study comparing 7 days vs. 14 days of antimicrobial therapy to treat bacteremia?
  - Yes--regardless of whether the line was removed
  - Yes--only if the line was removed
  - Yes--only if the line was not removed
  - No

**Patient 3**
A 17-month old female is transferred to the PICU from the Emergency Department with septic shock. She has had persistent fevers for the past 2 days. A blister is noted on her right foot, which parents say was from wearing a new pair of shoes a few days ago.

The patient is febrile, lethargic, tachycardic, hypotensive and has prolonged capillary refill time. She is given three IV boluses of 20 mL/kg normal saline and is started on norepinephrine. Blood and urine cultures are sent. The blister on the foot is de-roofed and a sample of serous liquid from the blister is sent to the microbiology lab. The patient is started on ceftriaxone and vancomycin.

At 24 hours, the patient is afebrile and has weaned off norepinephrine. The culture from the foot blister and her blood specimen are reported positive for Group A streptococcus. She is diagnosed with bloodstream infection secondary to a non-necrotizing soft tissue infection in her foot.

* 16. Based on the information above, what total duration (in days) of antibiotic therapy would you usually/typically recommend for this patient?
  - Number of days

* 17. Would you be willing to enrol a patient like this into a study comparing 7 days vs. 14 days of antimicrobial therapy to treat bacteremia?

- o Yes
- o No

**Patient 4**
A 6-week old male with a history of jejunal atresia and small bowel resection is admitted to the PICU post-operatively after an anastomosis repair to relieve recurrent bowel obstruction.

Over the next 48 hours, the patient is febrile with temperatures persistently >38.5 C and tachycardic. Erythema is observed over the surgical site and his abdomen is firm and distended. An abdominal ultrasound shows echogenic fluid in the left lower quadrant extending to the midline and right upper quadrant.

The patient's abdomen is re-opened for a washout and a drain is inserted. The fluid from the drain is seropurulent and sent to the microbiology lab. Blood cultures are also sent. The patient is started on meropenem and vancomycin.

At 24 hours, the abdominal fluid and blood cultures are reported positive for *Enterobacter cloacae*. He is diagnosed with bloodstream infection secondary to an intra-abdominal infection.

* 18. Based on the information above, what total duration (in days) of antibiotic therapy would you usually/typically recommend for this patient?

- o Number of days

* 19. Suppose that the abdominal fluid could not be drained or could only be partially drained. What approximate duration (in days) of antibiotic therapy would you usually/total typically recommend?

- o Number of days

* 20. If the fluid collection is completely drained, would you be willing to enrol a patient like this into a study comparing 7 days vs. 14 days of antimicrobial therapy to treat bacteremia?

- o Yes
- o No

**Patient 5**
A 10-year old female presents to the Emergency Department with fever, tachycardia, hypotension, progressive vomiting and looking unwell. She is given three IV boluses of 20 mL/kg normal saline and is started on vasopressors. Bloodwork is ordered and blood and urine cultures are drawn. She is empirically started on ceftriaxone and vancomycin and transferred to the pediatric intensive care unit for further management.

At 24 hours, the patient has significantly improved and is weaned off vasopressors. Her blood and urine cultures are reported positive for *Klebsiella pneumoniae*. She is diagnosed with bloodstream infection secondary to a urinary tract infection.

* 21. Based on the information above, what total duration (in days) of antibiotic therapy would you usually/typically recommend for this patient?

- o Number of days

* 22. Would you be willing to enrol a patient like this into a study comparing 7 days vs. 14 days of antimicrobial therapy to treat bacteremia?

- Yes
- No

**Extrapolating data from an adult trial**

* 23. Assume a well-designed randomized controlled trial in 3600 adult critically ill patients has been published which compared mortality among adult patients receiving 7 days vs. 14 days of antibiotic treatment for bacteremia. This trial demonstrated that the mortality rate in those receiving 7 days of therapy was no worse (non-inferior) than those who received 14 days.

Based on this adult study, would you be willing to prescribe 7 days of treatment for your pediatric critically ill patients with similar types of bloodstream infections?

- No, definitely not
- No, unlikely
- Yes, probably
- Yes, definitely

**Pediatric outcomes**

* 25. If insufficient resources are available for a pediatric trial powered for a mortality outcome, which of the following outcomes would be important enough as a primary outcome to guide your antibiotic treatment practices for pediatric ICU patients with bloodstream infections. (Check all that apply)

- Time to clinical stability
- Duration of mechanical ventilation
- Measures of organ dysfunction
- Length of ICU stay
- Length of hospital stay
- Neurodevelopmental outcomes
- Functional status outcomes
- Measures of frailty
- Quality of life
- None of the above
- Other (please specify)

**Thank you!**

Thank you very much for taking time to complete this survey! Your participation is very much appreciated and will contribute to understanding common durations of antibiotic therapy for bloodstream infections in critically ill children and the interpretation and choice of clinical outcomes that critical care and infectious diseases clinicians like yourself, consider important in these patients.

If you have any questions or concerns about this survey, please contact Sandy Pong by email (sandra.pong@sickkids.ca).

**Notes**

[1]The survey question on interpretation of pediatric trial results was analyzed and reported separately.

[2]Respondents were able to review and change their answers with a 'Back' button provided in the survey. Surveys with some questions unanswered were included in analyses.

[3]Same IP addresses were allowed if the database entries were different or made at separate dates or time (ie. 24 hours). When there were duplicate identical database entries with the same IP address within 24 hours, the most recent was kept for analysis.
