# Supplementary material for: Antibiotic treatment duration for bloodstream infections in critically ill children—A survey of pediatric infectious diseases and critical care clinicians for clinical equipoise
Source: PLoS One. 2022 Jul 26;17(7):e0272021. doi: 10.1371/journal.pone.0272021 (PMC9321425; doi:10.1371/journal.pone.0272021)
Supplement: S1 Table — (DOCX) [file pone.0272021.s002.docx]

**Supplement Table 1. Median (IQR) treatment duration (days) by clinician specialty.**

|  | Number (n) | Median (IQR) (days) | p-value*^a,b^* |
| --- | --- | --- | --- |
| Pneumonia | | | |
| Critical care  Infectious diseases  Pharmacy | 76  35  18 | 10 (7-10)  10 (10-10)  8.5 (7-10) | 0.6 |
| Skin/soft tissue | | | |
| Critical care  Infectious diseases  Pharmacy | 66  29  13 | 10 (7-14)  10 (10-14)  14 (10-14) | 0.12 |
| Urinary tract | | | |
| Critical care  Infectious diseases  Pharmacy | 65  29  13 | 10 (7-14)  10 (10-14)  10 (7-10) | 0.46 |
| Intra-abdominal (drained) | | | |
| Critical care  Infectious diseases  Pharmacy | 66  29  13 | 14 (14-14)  14 (10-14)  14 (7-14) | 0.11 |
| Intra-abdominal (partial/no drain) | | | |
| Critical care  Infectious diseases  Pharmacy | 66  29  13 | 21 (14-21)  21 (14-21)  14 (14-21) | 0.37 |

*^a^*Kruskal-Wallis Test

*^b^*Bonferroni adjusted p-value threshold = 0.02
