# Supplementary material for: Antibiotic treatment duration for bloodstream infections in critically ill children—A survey of pediatric infectious diseases and critical care clinicians for clinical equipoise
Source: PLoS One. 2022 Jul 26;17(7):e0272021. doi: 10.1371/journal.pone.0272021 (PMC9321425; doi:10.1371/journal.pone.0272021)
Supplement: S2 Table — (DOCX) [file pone.0272021.s003.docx]

**Supplement Table 2a. Median (IQR) treatment duration (days) by years since graduation.**

|  | Number (n) | Median (IQR) (days) | p-value*^a,b^* |
| --- | --- | --- | --- |
| Pneumonia | | | |
| 0-5 years  6-10 years  11-15 years  16-20 years  ≥21 years | 10  29  31  29  30 | 8.5 (7-10)  10 (7-14)  10 (7-10)  10 (7-10)  10 (7-10) | 0.42 |
| Skin/soft tissue | | | |
| 0-5 years  6-10 years  11-15 years  16-20 years  ≥21 years | 6  24  25  24  29 | 12 (10-14)  10 (7-14)  10 (7-14)  10 (10-14)  10 (10-14) | 0.63 |
| Urinary tract | | | |
| 0-5 years  6-10 years  11-15 years  16-20 years  ≥21 years | 6  23  25  24  29 | 8.5 (7-10)  10 (7-14)  10 (7-10)  10 (7-14)  10 (7-10) | 0.71 |
| Intra-abdominal (drained) | | | |
| 0-5 years  6-10 years  11-15 years  16-20 years  ≥21 years | 6  24  25  24  29 | 14 (10-14)  14 (12-14)  14 (10-14)  14 (14-14)  14 (14-14) | 0.4 |
| Intra-abdominal (partial/not drained) | | | |
| 0-5 years  6-10 years  11-15 years  16-20 years  ≥21 years | 6  24  25  24  29 | 17.5 (14-21)  14 (14-21)  14 (14-21)  21 (14-21)  21 (14-21) | 0.89 |

^a^Kruskal-Wallis Test

*^b^*Bonferroni adjusted p-value threshold = 0.005

**Supplement Table 2b. Median (IQR) treatment duration (days) of central vascular catheter-associated bacteremia by years since graduation.**

|  | 0-5 years  (n=6) | 6-10 years (n=24) | 11-15 years (n=25) | 16-20 years (n=26) | ≥21 years (n=29) | p-value*^a,b^* |
| --- | --- | --- | --- | --- | --- | --- |
| Catheter removed (n=110) | | | | | | |
| *Enterococcus faecalis*  *Staphylococcus aureus*  *Klebsiella pneumoniae*  Coagulase negative staphylococci  *Escherichia coli*  *Enterobacter cloacae*  *Pseudomonas aeruginosa* | 10 (7-10)  14 (14-14)  10 (10-14)  7 (5-10)  10 (10-14)  10 (10-14)  14 (14-14) | 10 (7-10)  10 (7-14)  10 (7-14)  7 (5-7)  10 (7-14)  10 (7-14)  14 (8.5-14) | 7 (7-10)  10 (7-14)  10 (7-10)  7 (5-10)  10 (7-14)  10 (7-14)  10 (7-14) | 7 (7-10)  14 (7-14)  10 (10-14)  7 (7-14)  10 (10-14)  10 (10-14)  14 (10-14) | 7 (7-10)  10 (7-14)  10 (7-10)  7 (5-10)  10 (7-10)  10 (7-10)  10 (10-14) | 0.79  0.15  0.14  0.44  0.45  0.3  0.04 |
| Catheter not removed (n=109) | | | | | | |
| *Enterococcus faecalis*  *Staphylococcus aureus*  *Klebsiella pneumoniae*  Coagulase negative staphylococci  *Escherichia coli*  *Enterobacter cloacae*  *Pseudomonas aeruginosa* | 14 (14-14)  17.5 (14-28)  14 (14-14)  12 (10-14)  14 (14-14)  14 (14-14)  17.5 (14-21) | 14 (14-14)  14 (14-21)  14 (14-14)  14 (14-14)  14 (14-14)  14 (14-14)  14 (14-17.5) | 14 (10-14)*^c^*  14 (14-17.5*^c^*  14 (12-14)*^c^*  14 (7-14)*^c^*  14 (10-17.5)*^c^*  14 (12-17.5)*^c^*  14 (14-17.5)*^c^* | 14 (10-14)  14 (14-14)  14 (10-14)  14 (10-14)  14 (10-14)  14 (10-14)  14 (14-14) | 14 (10-14)  14 (10-14)  14 (10-14)  10 (10-14)  14 (14-14)  14 (14-14)  14 (14-14) | 0.07  0.04  0.11  0.05  0.45  0.26  0.06 |

*^a^*Kruskal-Wallis Test

*^b^*Bonferroni adjusted p-value threshold = 0.005

*^c^*Missing = 1
