# Supplementary material for: Antibiotic treatment duration for bloodstream infections in critically ill children—A survey of pediatric infectious diseases and critical care clinicians for clinical equipoise
Source: PLoS One. 2022 Jul 26;17(7):e0272021. doi: 10.1371/journal.pone.0272021 (PMC9321425; doi:10.1371/journal.pone.0272021)
Supplement: S3 Table — (DOCX) [file pone.0272021.s004.docx]

**Supplement Table 3a. Median (IQR) treatment duration (days) by number of bloodstream infections managed per year.**

|  | Number (n) | Median (IQR) (days) | p-value*^a,b^* |
| --- | --- | --- | --- |
| Pneumonia | | | |
| 0-10 per year  11-20 per year  ≥21 per year | 15  48  66 | 10 (10-14)  10 (7-10)  10 (7-10) | 0.23 |
| Skin/soft tissue | | | |
| 0-10 per year  11-20 per year  ≥21 per year | 8  45  55 | 12 (8.5-14)  10 (10-14)  10 (7-14) | 0.87 |
| Urinary tract | | | |
| 0-10 per year  11-20 per year  ≥21 per year | 8  45  54 | 12 (8.5-14)  10 (7-14)  10 (7-10) | 0.46 |
| Intra-abdominal (drained) | | | |
| 0-10 per year  11-20 per year  ≥21 per year | 8  45  55 | 14 (8.5-14)  14 (14-14)  14 (10-14) | 0.31 |
| Intra-abdominal (partial/not drained) | | | |
| 0-10 per year  11-20 per year  ≥21 per year | 8  45  55 | 21 (14-21)  21 (14-21)  14 (14-21) | 0.76 |

^a^Kruskal-Wallis Test

*^b^*Bonferroni adjusted p-value threshold = 0.02

**Supplement Table 3b. Median (IQR) treatment duration (days) of central vascular catheter-associated bacteremia by number of bloodstream infections managed per year.**

|  | 0-10 per year  (n=9) | 11-20 per year (n=46) | ≥21 per year (n=55) | p-value*^a,b^* |
| --- | --- | --- | --- | --- |
| Catheter removed (n=110) | | | | |
| *Enterococcus faecalis*  *Staphylococcus aureus*  *Klebsiella pneumoniae*  Coagulase negative staphylococci  *Escherichia coli*  *Enterobacter cloacae*  *Pseudomonas aeruginosa* | 7 (7-14)  10 (7-14)  10 (7-14)  7 (7-10)  10 (10-14)  14 (10-14)  14 (10-14) | 10 (7-10)  10 (7-14)  10 (7-10)  7 (7-10)  10 (7-14)  10 (10-14)  14 (10-14) | 7 (7-10)  10 (7-14)  10 (7-14)  7 (5-7)  10 (7-14)  10 (7-14)  10 (7-14) | 0.03  0.95  0.69  0.01  0.5  0.21  0.18 |
| Catheter not removed (n=109) | | | | |
| *Enterococcus faecalis*  *Staphylococcus aureus*  *Klebsiella pneumoniae*  Coagulase negative staphylococci  *Escherichia coli*  *Enterobacter cloacae*  *Pseudomonas aeruginosa* | 14 (14-14)  14 (14-28)  14 (14-14)  14 (14-14)  14 (14-14)  14 (14-14)  14 (14-14) | 14 (14-14)*^c^*  14 (14-14)*^c^*  14 (14-14)*^c^*  14 (10-14)*^c^*  14 (14-14)*^c^*  14 (14-14)*^c^*  14 (14-14)*^c^* | 14 (10-14)  14 (14-14)  14 (14-14)  10 (7-14)  14 (10-14)  14 (14-14)  14 (14-14) | 0.01  0.54  0.71  0.04  0.36  0.35  0.8 |

*^a^*Kruskal-Wallis Test

*^b^*Bonferroni adjusted p-value threshold = 0.02

*^c^*Missing = 1
