# Supplementary material for: Antibiotic treatment duration for bloodstream infections in critically ill children—A survey of pediatric infectious diseases and critical care clinicians for clinical equipoise
Source: PLoS One. 2022 Jul 26;17(7):e0272021. doi: 10.1371/journal.pone.0272021 (PMC9321425; doi:10.1371/journal.pone.0272021)
Supplement: S4 Table — (DOCX) [file pone.0272021.s005.docx]

**Supplement Table 4a. Median (IQR) treatment duration (days) by presence of institutional antimicrobial stewardship program.**

|  | Number (n) | Median (IQR) (days) | p-value*^a^* |
| --- | --- | --- | --- |
| Pneumonia | | | |
| Antimicrobial stewardship program  No antimicrobial stewardship program | 97  32 | 10 (7-10)  10 (7-14) | 0.54 |
| Skin/soft tissue | | | |
| Antimicrobial stewardship program  No antimicrobial stewardship program | 84  24 | 10 (10-14)  10 (7-14) | 0.62 |
| Urinary tract | | | |
| Antimicrobial stewardship program  No antimicrobial stewardship program | 83  24 | 10 (7-14)  10 (7-14) | 0.36 |
| Intra-abdominal (drained) | | | |
| Antimicrobial stewardship program  No antimicrobial stewardship program | 84  24 | 14 (10-14)  14 (14-14) | 0.26 |
| Intra-abdominal (partial/not drained) | | | |
| Antimicrobial stewardship program  No antimicrobial stewardship program | 84  24 | 14 (14-21)  21 (14-21) | 0.58 |

^a^Wilcoxon Rank Sum Test

**Supplement Table 4b. Median (IQR) treatment duration (days) of central vascular catheter-associated bacteremia by presence of institutional antimicrobial stewardship program.**

|  | Antimicrobial stewardship program (n=85) | No antimicrobial stewardship program (n=25) | p-value*^a^* |
| --- | --- | --- | --- |
| Catheter removed (n=110) | | | |
| *Enterococcus faecalis*  *Staphylococcus aureus*  *Klebsiella pneumoniae*  Coagulase negative staphylococci  *Escherichia coli*  *Enterobacter cloacae*  *Pseudomonas aeruginosa* | 7 (7-10)  10 (7-14)  10 (7-10)  7 (5-10)  10 (7-14)  10 (7-14)  10 (7-14) | 10 (10-14)  14 (7-14)  10 (7-14)  7 (7-10)  10 (10-14)  10 (10-14)  14 (10-14) | 0.003  0.5  0.25  0.12  0.25  0.13  0.07 |
| Catheter not removed (n=109) | | | |
| *Enterococcus faecalis*  *Staphylococcus aureus*  *Klebsiella pneumoniae*  Coagulase negative staphylococci  *Escherichia coli*  *Enterobacter cloacae*  *Pseudomonas aeruginosa* | 14 (10-14)*^b^*  14 (14-14)*^b^*  14 (14-14)*^b^*  14 (10-14)*^b^*  14 (14-14)*^b^*  14 (14-14)*^b^*  14 (14-14)*^b^* | 14 (14-14)  14 (14-21)  14 (14-14)  14 (10-14)  14 (14-14)  14 (14-14)  14 (14-21) | 0.17  0.5  0.6  0.3  0.96  0.45  0.42 |

*^a^*Kruskal-Wallis Test

*^b^*Missing = 1
