# Supplementary material for: Antibiotic treatment duration for bloodstream infections in critically ill children—A survey of pediatric infectious diseases and critical care clinicians for clinical equipoise
Source: PLoS One. 2022 Jul 26;17(7):e0272021. doi: 10.1371/journal.pone.0272021 (PMC9321425; doi:10.1371/journal.pone.0272021)
Supplement: S5 Table — (DOCX) [file pone.0272021.s006.docx]

**Supplement Table 5. Respondents who were willing to enrol patients into a trial of 7 versus 14 days of antimicrobial therapy and recommended at least 10 days of antimicrobial therapy for central vascular catheter-associated infections in case scenarios.**

| Organism | Catheter removed (n=105) | Catheter not removed (n=104) |
| --- | --- | --- |
| *Enterococcus faecalis*  *Staphylococcus aureus*  *Klebsiella pneumoniae*  Coagulase negative staphylococci  *Escherichia coli*  *Enterobacter cloacae*  *Pseudomonas aeruginosa* | 48 (46%)  68 (65%)  66 (63%)  30 (29%)  71 (68%)  73 (70%)  81 (77%) | 95 (91%)  95 (91%)  96 (92%)  81 (78%)  99 (95%)  100 (96%)  101 (97%) |
