# Supplementary material for: Antibiotic treatment duration for bloodstream infections in critically ill children—A survey of pediatric infectious diseases and critical care clinicians for clinical equipoise
Source: PLoS One. 2022 Jul 26;17(7):e0272021. doi: 10.1371/journal.pone.0272021 (PMC9321425; doi:10.1371/journal.pone.0272021)
Supplement: S6 Table — (DOCX) [file pone.0272021.s007.docx]

**Supplement Table 6. Comparison of respondents recommending longer (≥10 days) treatment durations between infectious syndromes.**

| Syndrome | Number of respondents*^a^* (n) | Recommend longer treatment (>=10 days), n (%) | Pneumonia | Skin/soft tissue | Urinary tract | Intra-abdominal (not drained) | Intra-abdominal (not drained) | Central vascular catheter-associated infection due to *E. coli* (catheter not removed) | Central vascular catheter-associated infection due to *E. coli* (catheter removed) |
| --- | --- | --- | --- | --- | --- | --- | --- | --- | --- |
| Pneumonia | 129 | 84 (65) | -- |  |  |  |  |  |  |
| Skin/soft tissue | 108 | 80 (74) | <0.0001*^b^* | -- |  |  |  |  |  |
| Urinary tract | 107 | 68 (64) | 0.0006*^b^* | 0.59*^b^* | -- |  |  |  |  |
| Intra-abdominal (drained) | 108 | 97 (90) | 0.11*^c^* | 0.47*^c^* | 0.09*^c^* | -- |  |  |  |
| Intra-abdominal (not drained) | 108 | 107 (99) | 1*^c^* | 1*^c^* | 1*^c^* | 1*^c^* | -- |  |  |
| Central vascular catheter-associated infection due to *E. coli* (catheter not removed)*^c^* | 109 | 104 (95) | 0.08*^c^* | 0.11*^c^* | 0.05*^c^* | 0.08*^c^* | 1*^c^* | -- |  |
| Central vascular catheter-associated infection due to *E. coli* (catheter removed)*^d^* | 110 | 76 (69) | 0.02*^b^* | 0.13*^b^* | 0.0002*^b^* | 0.03*^c^* | 0.31*^c^* | 0.03*^c^* | -- |

*^a^*Number of respondents differ because some respondents did not answer every question

*^b^*Chi-square Test

*^c^*Fisher’s Exact Test

*^d^E. coli* bacteremia selected as an example of a central vascular catheter-associated infection to compare with other infectious syndromes
